# Patient preference survey: are patients willing to delay surgery if obstructive sleep apnea is suspected?

**DOI:** 10.1186/s12871-018-0594-5

**Published:** 2018-09-12

**Authors:** George Ho, Crispiana Cozowicz, Jean Wong, Mandeep Singh, Enoch Lam, Eva E. Mörwald, Najia Hasan, Stavros G. Memtsoudis, Frances Chung

**Affiliations:** 1 Department of Anesthesiology and Pain Medicine, Toronto Western Hospital, University Health Network, University of Toronto, Toronto, ON Canada; 2 Faculty of Medicine, University of Toronto, Toronto, ON Canada; 3 Department of Anesthesiology, Hospital for Special Surgery, Weill Cornell Medical College, New York, NY USA; 4 Perioperative Medicine and Intensive Care Medicine, Paracelsus Medical University, Salzburg, Austria; 5 Toronto Sleep and Pulmonary Centre, Toronto, ON Canada; 6 Perioperative Medicine and Intensive Care Medicine, Paracelsus Medical University, Salzburg, Austria

**Keywords:** Obstructive sleep apnea, Anesthesiology, Surveys, Decision making

## Abstract

**Background:**

Screening and optimizing patients for OSA in the perioperative period may reduce postoperative complications. However, sleep studies can be difficult to obtain before surgery. Previous surveys reported that the majority of sleep physicians would delay surgery to diagnose and manage OSA, but most anesthesiologists would not. While disagreements exist, the importance of shared decision making and patient preferences have never been studied on this topic. It is unknown whether patients with suspected OSA, when given information about OSA, would be willing to delay surgery to diagnose and manage their condition preoperatively.

**Methods:**

This study consisted of a self-administered questionnaire that surveyed patients, patient relatives, or any accompanying members. The survey was conducted in the preoperative clinic or in the perioperative patient and family waiting area at two hospitals in Canada and in the United States. A hypothetical scenario was used: participants were given information about OSA, and asked about their preferences regarding preoperative management should they be at risk for OSA in the setting of pending elective surgery. The objective of this study was to determine whether respondents preferred to 1) proceed with surgery as planned, 2) delay surgery to ensure the medical condition of OSA is diagnosed and optimized, or 3) let his/her physician decide.

**Results:**

The final survey contained 19 questions and the survey was conducted from June 2016 to September 2016. Four hundred and seventy-three surveys were collected. Forty-four percent of respondents, when given information about OSA, preferred to delay surgery pending a sleep study and treatment. Forty percent of respondents who preferred to delay surgery would tolerate delaying up to two months.

**Conclusion:**

Increasing emphasis and significant value has been placed on shared-decision making between patients and physicians. Educating patients about the risks of OSA and incorporating patient preferences into the perioperative management of OSA may be warranted.

**Electronic supplementary material:**

The online version of this article (10.1186/s12871-018-0594-5) contains supplementary material, which is available to authorized users.

## Background

Obstructive sleep apnea (OSA) is the most common sleep breathing disorder. The prevalence of OSA in the general population is estimated to be 10–20% [1]. Surgical patients with OSA have an increased risk of postoperative complications, thus identifying OSA patients prior to surgery may ensure optimal perioperative care [2, 3].

There exists significant variation in opinion and practice among anesthesiologists and sleep physicians with respect to the preoperative evaluation and optimization of patients suspected of having OSA. Two surveys conducted amongst anesthesiologists reported that only 25% would delay surgery pending a sleep study to ensure proper diagnosis and treatment if a patient was suspected of having OSA [4, 5]. Another recent questionnaire conducted among four specialties (anesthesiology, primary care, sleep medicine and general surgery), concluded that a mere 4% of anesthesiologists would delay surgery pending a sleep study if a patient was suspected of having OSA, while 22% would simply proceed with surgery [6]. Conversely, among sleep physicians, 54% would prefer to delay surgery and only 5% would proceed with surgery [6].

Since patient preferences have not been studied on this topic, it is unclear whether patients, if suspected to have OSA, would prefer to proceed with or delay surgery pending a sleep study and treatment. Moreover, since 2001 the Institute of Medicine has recognized that adopting patient-centered care is crucial to the improvement of the health care system [7], and the Canadian healthcare system has also placed significant interest and high value on shared decision making that encompasses patient-centered care [8]. Recommendations and treatment should align with patient values and needs, given that it is ultimately the patient who must make informed choices about his or her care [9, 10]. The purpose of this survey was to, for the first time, examine participants’ preferences by questioning participants regarding their preferences should they be suspected with having OSA while awaiting elective surgery.

## Methods

### Study design

This study surveyed patients, patient relatives, or any accompanying members in the preoperative clinic at Toronto Western Hospital (TWH), Toronto, and in the perioperative patient and family waiting area at the Hospital for Special Surgery (HSS), New York, using a self-administered questionnaire. As per the recommendation of the institutional ethics boards, to avoid unnecessary stress before surgery, we did not specifically target patients diagnosed with OSA. Approval from the Research Ethics Board/Institutional Review Board at TWH and HSS was obtained, and written informed consent was waived. Completion of the survey implied consent. The types of surgery performed at TWH include orthopedic, spine, neurosurgery, general surgery, urology, ophthalmology, and hand surgery. The types of surgery performed at HSS include inpatient orthopedic and spine surgery. TWH is a publicly funded healthcare facility, and HSS is a private but not-for-profit healthcare facility.

### Primary and secondary objectives

The primary objective of our survey was to determine the preferences of participants when presented with information regarding OSA and a hypothetical scenario in which they are patients scheduled for elective surgery, but are suspected of having OSA. More specifically, which option would participants prefer the most: 1) proceed with surgery as planned, 2) delay surgery to ensure the medical condition of OSA is diagnosed and optimized before surgery, or 3) let his/her physician decide the course of action.

The secondary objectives included: 1) testing the knowledge of participants on OSA (symptoms, risks), 2) inquiry of current treatments amongst participants with OSA, 3) determination of the maximum length of delay that would be tolerated, if respondents preferred to delay surgery, and 4) when making a treatment decision, which domains do participants value the most [e.g. shortest length of stay, lowest risk of intensive care unit (ICU) transfers, etc.].

### Survey development

A literature search of PubMed was conducted and no prior surveys investigating the topic of patient preferences presenting for elective surgery with suspected OSA were found. Due to the lack of prior investigations on this topic, content experts were enlisted to develop the survey. Anesthesiologists and sleep physicians were the content experts (FC, SM, CC, NH, MS, and JW) who identified potentially important key categories and themes for evaluation. A list of items was generated from content expert interviews and expanded with input from all of the investigators.

### Domains and items reduction

Content experts ranked the importance of each domain and item, and the following domains emerged: knowledge of OSA including OSA diagnosis and current treatment for participants with OSA; when given information regarding OSA and the postoperative risks, do participants understand the information; if given a hypothetical scenario: proceed or delay surgery; participant values ranking: shortest length of hospital stay, risk of complications during surgery, risk of postoperative complications, risk of ICU transfer; and involvement in decision making process with his or her physician.

### Survey formatting

The survey consisted of four parts:

- General socio-demographics of survey participants.
- General understanding of OSA, OSA diagnosis, and current treatment for participants with OSA.
- Presentation of information on OSA and the postoperative risks associated with OSA. The information was presented on one page titled: “Part 3: The following is information about Obstructive Sleep Apnea”, and was described in 4 paragraphs. Paragraph 1 explained what OSA is, the symptoms associated with OSA, and the possible consequences of not treating the condition. Paragraph 2 explained what a sleep study is and how it is conducted at a sleep clinic. Paragraph 3 explained possible management modalities (oral appliances, breathing devices, surgery) if a patient has been diagnosed with OSA. Paragraph 4 explained that in the context of OSA patients undergoing surgery, they may be at an increased risk of postoperative complications. Participants were informed: “Compared to surgical patients without sleep apnea, patients with obstructive sleep apnea are:
  At close to **2.5 times** greater risk of developing breathing problems after surgery
  At more than **1.5 times greater** risk of developing heart problems after surgery.”

Participants were then asked to answer 3 follow-up questions at the bottom of the page so that we could assess participant understanding of the presented information.

3b) Presentation of a hypothetical scenario to evaluate whether participants prefer to delay surgery, proceed with surgery, or let his or her physician decide. Also, for the participants who prefer to delay surgery, the maximum length of delay that would be tolerated. The lengths of delay (2 weeks, 1 month and 2 months) were determined based on a survey of family physicians, ear, nose and throat physicians, and respirologists in Ontario, Canada which reported that the most common response for wait time to undergo a sleep study was 1 to 3 months [11].
4) Five questions were asked to evaluate characteristics participants value the most during the decision making process (e.g. length of hospital stay, risk of intraoperative complications, risk of postoperative complications, risk of ICU transfer, and involvement in the decision making process with his or her physician.

### Survey pre/pilot testing

The draft survey was distributed to the investigators who revised the study instrument focusing on clarity and interpretation of the questions. We pilot-tested the survey on ten volunteers from a variety of educational backgrounds. Revisions were made to improve language, and overall clarity of the survey (Additional file 1).

### Implementation of the study

Study posters and information were posted in the preoperative clinic at TWH, and in the perioperative waiting area at HSS, while surveys were placed on tables in the respective areas for completion. A response box was set up beside the surveys allowing the study participants to anonymously deposit their completed surveys. Potential participants could pick up the survey, read through the instructions provided on the cover sheet, and complete the survey voluntarily.

The cover sheet indicated that completion of the survey and submission of the survey into the provided response box implied that he or she had consented to participate in the study. Furthermore, the cover sheet instructed respondents to complete the survey in the order that the questions were given, and respondents were asked to answer all questions to the best of their knowledge. Research assistants and other hospital staff informed individuals waiting in the respective clinic areas about the opportunity to complete a survey. As well, the research assistants were available at both sites to answer any questions.

Study participants completed the survey either on the day of the preoperative clinic appointment at TWH, or on the day of the operation at HSS. Both institutions screen patients for OSA, but the surveys were completed when patients were in the waiting room before his/her appointment with nurses and anesthesiologists. Each survey was assigned a coded study identification number. The respondents had the option to skip any question(s) they did not wish to answer. The Table 1 indicates the total number of respondents at both sites, as well as, the total number of responses obtained for each survey question.

**Table 1:** Summary of the response rates and responses to the survey questions by site

Survey Question	Total *n* (% of total responses)	HSS *n* (% of total responses)	TWH n (% of total responses)	*P*-value
Gender, male/female^a^ (T:473, HSS:244, TWH:229)				0.22
Male	187 (40)	103 (42)	84 (37)	
Female	286 (60)	141 (58)	145 (63)	
Age, years^b^ (T:405, HSS:181, TWH:224)	55 ± 16	53 ± 17	56 ± 16	0.07
Highest educational attainment^a^ (T:473, HSS:244, TWH:229)				&lt; 0.001‡
Less than high school/secondary school	15 (3)	1 (0)	14 (6)	
High school (or equivalent)	88 (19)	33 (14)	55 (24)	
College/University (diploma and/or degree)	365 (77)	208 (85)	157 (69)	
Prefer not to provide this information	5 (1)	2 (1)	3 (1)	
Familiar with sleep apnoea?^a^ (T:472, HSS:244, TWH:228)				0.03‡
Yes	421 (89)	225 (92)	196 (86)	
No	51 (11)	19 (8)	32 (14)	
Diagnosed with OSA?^a^ (T:430, HSS:224, TWH:206)				0.44
Yes	55 (13)	26 (12)	29 (14)	
No	375 (87)	198 (88)	177 (86)	
If diagnosed with OSA, the current treatment being used^a^ (T:55, HSS:26, TWH:29)				1.00
Use CPAP nightly	30 (55)	14 (54)	16 (55)	
Use CPAP sometimes	2 (4)	1 (4)	1 (3)	
Does not use CPAP	4 (7)	2 (8)	2 (7)	
Other treatment (Oral appliances, surgery)	4 (7)	2 (8)	2 (7)	
No treatment	15 (27)	7 (27)	8 (28)	
Participants’ knowledge of OSA symptoms^a^ (T:469, HSS:242, TWH:227)				
Short episodes where you stop breathing whilst asleep	386 (82)	207 (85)	179 (79)	&lt; 0.001‡
Feeling very tired during the daytime	312 (67)	164 (68)	148 (65)	0.56
Sudden awakening during sleep with choking or gasping	311 (66)	164 (68)	147 (65)	0.49
Loud persistent snoring	306 (65)	167 (69)	139 (61)	0.08
*Checked off all four of the above symptoms*	208 (44)	112 (46)	96 (42)	0.38
OSA may affect your long-term health^a^ (T:470, HSS:243, TWH:227)				0.16
True	430 (91)	227 (93)	203 (89)	
False	11 (2)	6 (2)	5 (2)	
Cannot decide	29 (6)	10 (4)	22 (8)	
OSA is treatable by different therapies^a^ (T:469, HSS:243,TWH:226)				0.02‡
True	426 (91)	222 (91)	204 (90)	
False	6 (1)	6 (2)	0	
Cannot decide	37 (8)	15 (6)	22 (10)	
Hypothetical Scenario – Comprehension Questions^a^ (T:467, HSS:241, TWH:226)				&lt; 0.001‡
All 3 questions correct	344 (74)	188 (78)	156 (69)	
2/3 questions correct	92 (19)	46 (19)	46 (20)	
1 question correct	19 (4)	7 (3)	12 (5)	
0 questions correct	3 (1)	0	3 (1)	
“Cannot Decide” for all 3 questions	9 (2)	0	9 (4)	
Hypothetical Scenario – Proceed/Delay/Physician Decides^a^ (T:453, HSS:237, TWH:216)				&lt; 0.001‡
Proceed with surgery	111 (25)	47 (19)	64 (29)	
Delay surgery	201 (44)	126 (53)	75 (35)	
Let my physician decide	141 (31)	64 (27)	77 (36)	
If “Yes” to Delaying Surgery: length of delay tolerated^a^ (T:192, HSS:122, TWH:70)				0.41
Up to 2 weeks	49 (26)	34 (28)	15 (21)	
Up to 1 month	66 (34)	38 (31)	28 (40)	
Up to 2 months	77 (40)	50 (41)	27 (39)	
When making a decision as to which method of treatment you most prefer, how do you rate the importance of the following?^b^*(1 = least important, 5 = most important)* (T:448, HSS: 235, TWH: 212)	Mean Rating (Total)	Mean Rating (HSS)	Mean Rating (TWH)	*P*-value
Lowest risk of complications during surgery	4.74 ± 0.7	4.82 ± 0.6	4.67 ± 0.7	0.02‡
Lowest risk of postoperative complications	4.70 ± 0.7	4.82 ± 0.6	4.57 ± 0.8	&lt; 0.001‡
Lowest risk of being transferred to ICU	4.61 ± 0.9	4.65 ± 0.8	4.57 ± 0.9	0.33
Being involved in decision making process	4.46 ± 0.9	4.48 ± 0.9	4.44 ± 0.9	0.67
Shortest length of stay at the hospital	3.64 ± 1.3	3.69 ± 1.2	3.57 ± 1.3	0.33

CPAP = Continuous Positive Airway Pressure; HSS = Hospital for Special Surgery; ICU = Intensive Care Unit; OSA = Obstructive Sleep Apnea; T = total number of responses; TWH = Toronto Western Hospital

^a^Data presented as frequency (%)

^b^Data presented as mean ± SD and the Student Independent 2-sample t-test was used to check the statistical significance

‡Statistical significance (*P* &lt; 0.05)

### Statistical analysis

No estimation of sample size was done, as this was the first study to determine participants’ preferences on sleep disorders and treatment before surgery. The target was 500 completed surveys with 250 at each study site. We described the participants’ responses using percentages. Statistical analysis was performed between the following subgroups: site (TWH vs. HSS), sex (M vs. F), age (&lt; 65 years old vs. ≥ 65 years old), education level (secondary school education or less vs. college/university education), and respondents indicating they have OSA vs. respondents without OSA (OSA vs. non-OSA). Student’s t-tests for continuous variables and chi-square tests for categorical variables were used. *P* value &lt; 0.05 was considered significant.

## Results

The final survey contained 19 questions and the survey was conducted at TWH and HSS from June 2016 to September 2016. Of the 500 distributed surveys, 473 completed surveys were collected. The socio-demographics and response rates of the survey respondents are shown in the Table 1.

Eighty-nine percent (421/472) of respondents were familiar with “Sleep Apnea” and 13% (55/430) of respondents indicated having been diagnosed with OSA. Among the 55 respondents with OSA, 55% (30/55) reported using continuous positive airway pressure (CPAP) nightly, and 27% (15/55) disclosed that they were currently not on any treatment.

For questions 1–3 in Part 3, when given information about OSA, 74% (344/467) of respondents answered all 3 follow-up questions correctly. Nineteen percent (92/467) of respondents answered 2 out of the 3 questions correctly. When given the hypothetical scenario, 44% (201/453) of respondents indicated that he or she would delay surgery to ensure his/her OSA condition was treated and optimized prior to the surgery. Twenty-five percent (111/453) indicated that they would prefer to proceed with the surgery as planned, and 31% (141/453) indicated that they would prefer to let his/her physician make a decision regarding the plan of action. Of those who preferred to delay surgery, 40% (77/192) agreed to delay up to 2 months, pending a sleep study and treatment. If a participant checked yes to all three lengths of delay (2 weeks, 1 month or 2 months delay), it was inferred that the participant would agree to delay surgery for up to two months.

The difference in education levels between the two study sites was significant. Most notably, a higher proportion of respondents at HSS had a College/University education (TWH vs. HSS: 69% vs. 85%). Furthermore, there was a significant difference in the percentage of respondents who would delay surgery between both study sites (*P* &lt;  0.001). Fifty-three percent of respondents at HSS indicated they would prefer to delay surgery. In contrast, only 35% of respondents at TWH indicated they would prefer to delay surgery. A higher percentage of respondents at TWH would prefer to simply proceed with surgery as planned (TWH vs. HSS: 29% vs. 19%).

There was a significant difference in respondents who had secondary school education or less vs. respondents who had college/university education (*p* = 0.004). The same proportion of respondents in both groups would prefer to delay surgery (22%). A lower proportion of respondents with secondary school education or less would prefer to proceed with surgery (secondary school education or less vs. college/university education: 34% vs. 51%). A higher proportion of respondents with secondary school education or less would prefer to let his/her physician decide (secondary school education or less vs. college/university education: 44% vs. 28%).

There was a significant difference between respondents indicating they have OSA vs. respondents without OSA (*p* = 0.025). A higher proportion of respondents with OSA would prefer to proceed with surgery (OSA vs. non-OSA: 37% vs. 21%). A lower proportion of respondents with OSA would prefer to delay surgery (OSA vs. non-OSA: 31% vs. 48%). The same proportion of respondents in both groups would like his/her physician to decide (31%). There was no significant difference between sex (Male vs. Female), and age (&lt; 65 years old vs. ≥ 65 years old) regarding the delay of surgery.

For part 2 question 4 (“What do you believe are the likely symptoms of OSA”), 44% of respondents checked off all 4 symptoms as likely the result of OSA. There was no difference between the two study sites. For questions 5 and 6 (True/False), 91% of respondents believed OSA may affect long-term health, and is treatable by different therapies. There was no difference between the two sites on whether OSA may affect long-term health (question 5), but there was a significant difference on treatment by different therapies (question 6). For question 6, a higher proportion of respondents at TWH indicated they “Cannot decide” (TWH vs. HSS: 10% vs. 6%) and none of the respondents at TWH responded with “False” for this statement (*p* = 0.02).

## Discussion

The majority of respondents (89%) were already familiar with OSA as a medical condition. When given information on OSA, 93% were able to answer at least 2 out of the 3 follow-up questions correctly, demonstrating that respondents had a high level of understanding regarding OSA, i.e. how it is diagnosed, types of available treatment, and the associated postoperative risks. When presented with the hypothetical scenario of high risk for OSA and pending surgery, 44% indicated a preference that involved the delay of surgery to ensure that the medical condition of OSA was optimized prior to surgery. In this context, 40% of respondents were willing to tolerate a delay of up to 2 months.

Multiple guidelines have been published on the perioperative management of OSA patients [12–16]. For example, a recently published guideline by the American Society of Anesthesiologists recommends preoperative screening as well as initiation of pre-operative continuous positive airway pressure (CPAP) to optimize the OSA condition [14, 15]. However, access to diagnosis and treatment of OSA is limited, which means that a patient’s surgery may be delayed significantly if OSA is suspected and is postponed for further investigation [17]. In fact, the most recent Guideline by the Society of Anesthesia and Sleep Medicine notes that there is limited evidence to guide recommendations with respect to the preoperative decision-making process for patients and clinicians on whether to proceed or delay surgery if a patient is suspected of having OSA [16]. While disagreement exists between sleep physicians and anesthesiologists on whether a delay of surgery is warranted in patients suspected to have OSA [6], a paucity of data exists regarding patient preferences on this issue. At present, physicians have relied on their clinical experience and guidelines have not taken into account patient perspectives in the perioperative management of OSA.

Implementing shared decision making involves understanding patient preferences, while having an open and honest discussion with patients to ensure they are well informed [18]. A recently published article on the implementation of shared decision making in the National Health Service (NHS) recognizes that a change in attitude amongst physicians is key to improving the shared decision making process [18]. It reported that many physicians presume that they already involve their patients in care-related decisions, or physicians may fail to recognize how the values and preferences of patients may differ from their own. These findings have been recognized in Canada and the US, and are not unique to the NHS [10, 19]. In order to improve shared decision making, a true shift in attitude and culture is required [20]. However, none of this can be accomplished if clinicians do not investigate patient preferences or are not aware that patient perspectives may differ from their own perspectives.

OSA is a highly prevalent comorbid condition in the elective surgical population and preoperative identification and treatment of OSA may reduce perioperative complications. It is important for physicians to educate their patients about the potential risks and to involve patients in the decision making process and so it was necessary to investigate the preferences of patients. This study represents the first attempt to examine the preferences of participants using a hypothetical scenario, and demonstrates that a significant proportion of respondents would be willing to delay surgery pending a sleep study and treatment for OSA. Further investigations are warranted on more specific patient populations and the different types of surgical procedures. While an argument could be made that sleep studies and treatments may amount to greater short-term costs, the identification and treatment of OSA will result in reductions in overall healthcare costs in the long term [21, 22]. Anesthesiologists have the opportunity to become major contributors in shared decision making while diagnosing and treating OSA to improve perioperative safety in the healthcare system [17]. Recognition of patient preferences may assist decision making and supplement the formulation and/or modifications of guidelines or institutional policy for the perioperative management of patients with OSA.

There were limitations to this study with regards to the development, distribution and implementation of the survey. Validity testing was not conducted during development. We may have been able to obtain more fully completed surveys and illicit more accurate responses from participants if research assistants were available to guide each respondent through the survey in a one-on-one setting. Due to the nature of the survey, the type (patients and non-patients) and total number of participants attending the clinic or preoperative areas were not collected. The generalizability of the results to individuals with lower educational status may be limited, as most participants in this survey had completed a college/university education. Furthermore, individuals with previous knowledge on OSA may have been more likely to participate in the survey. Only a small portion of participants had OSA, which may have made the scenario more “real” and the data most reliable amongst this group. However, over half of such patients were already on treatment, so the question on delaying surgery would be of little relevance. We presented information on OSA and associated risks to ensure all respondents were informed of OSA, but the presentation of the risks may have biased respondents towards delaying surgery. We acknowledge that the hypothetical nature of the scenario removes respondents from real-life decision-making, and the type of procedure was not specified. The results may differ if respondents were asked to make a real-life decision on their upcoming surgery. The type of procedure may also significantly impact decision-making because elective surgery encompasses a wide range of procedures from cosmetic to cancer surgery. Moreover, when respondents were asked to rate the importance of several factors at the end of the survey, the inclusion of procedure types as a factor may have been of interest.

## Conclusions

Despite the limitations of this study, it was the first attempt to understand patient preferences within the preoperative management of suspected OSA. Our study demonstrates that patients do have clear preferences on this topic. Educating patients and incorporating shared decision making when facing a complex condition such as suspected OSA in the surgical setting may help physicians to make decisions about preoperative management of these patients.

## Additional file

Additional file 1: Survey on Participant Preference. (DOCX 28 kb)

## Abbreviations

CPAP: continuous positive airway pressure
HSS: Hospital for Special Surgery
ICU: intensive care unit
NHS: National Health Service
OSA: obstructive sleep apnea
TWH: Toronto Western Hospital
