# Supplementary material for: Patient preference survey: are patients willing to delay surgery if obstructive sleep apnea is suspected?
Source: BMC Anesthesiol. 2018 Sep 12;18:128. doi: 10.1186/s12871-018-0594-5 (PMC6136217; doi:10.1186/s12871-018-0594-5)
Supplement: Supplementary file 1 — Survey on Participant Preference. (DOCX 28 kb) [file 12871_2018_594_MOESM1_ESM.docx]

**Additional file 1 – Survey on Participant Preference**

**Part 1: Socio-demographics:**

1. Gender:

Male

Female

1. Age:       years
2. What is your highest educational attainment?

Less than high school/secondary school

High school (or equivalent)

College/University (Diploma and/or Degree)

Prefer not to provide this information

**Part 2: The following are questions on a medical condition called obstructive sleep apnea. Please respond to each question to the best of your knowledge**

1. Are you familiar with a medical condition called “**Sleep Apnea**”?

YES

NO

******If “NO”, please proceed to Question 4******

1. Have you been diagnosed with **Obstructive Sleep Apnea** at any point in your life (i.e. Done a sleep study at a sleep clinic and have diagnosis confirmed by a physician):

YES

NO

1. If answer to *Question 2* is “**YES”**:

I currently use Continuous Positive Airway Pressure (CPAP) nightly

I currently use CPAP sometimes, but not nightly

I currently do not use CPAP

I have received treatment in the form of oral appliances, and/or surgery for my obstructive sleep apnea (e.g. Nasal surgery, surgery for the jaw)

I am currently not receiving any treatment/therapy

1. What do you believe are the likely symptoms of obstructive sleep apnea?

*(You may check off more than one)*

Loud persistent snoring

Sudden awakening during sleep with choking or gasping

Short episodes where you stop breathing whilst asleep

Feeling very tired during the daytime

**Do you believe the two statements below are TRUE or FALSE?**

1. Obstructive sleep apnea may affect your long-term health (e.g. increase the risks of heart attacks, stroke, diabetes control and reduce life expectancy)

TRUE

FALSE

Cannot Decide

1. Obstructive sleep apnea is treatable by different therapies (e.g. weight loss, breathing devices such as CPAP, dental appliances, and in some people surgery)

TRUE

FALSE

Cannot Decide

**Part 3: The following is information about Obstructive Sleep Apnea.**

**Obstructive Sleep Apnea** is a common medical condition that affects breathing repeatedly during sleep. It causes brief episodes where the throat and airway collapses, blocking air from reaching the lungs. Those with this condition may experience sleepiness, tiredness and/or fatigue in the daytime. If left untreated, it may affect functioning of the lungs and heart. Furthermore, it can result in heart attacks, stroke, and decrease life expectancy in the long run.

Currently, a sleep study is the best way to diagnose obstructive sleep apnea but must be scheduled and conducted in a sleep center overnight. A sleep study measures your breathing and body functions overnight. The test is painless and measurements are made through sensors attached to your face, chest, scalp, and fingers.

Once a diagnosis is made, your doctor will determine the best treatment for management of this medical condition. Treatments may include lifestyle changes such as sleeping position. Other treatments include oral appliances, breathing devices or surgery. Continuous positive airway pressure (CPAP) machine is a breathing device that must be worn each night during sleep and is most commonly used for treatment.

Surgical patients under anesthesia with obstructive sleep apnea may be at an increased risk of complications after surgery. Compared to surgical patients without sleep apnea, patients with obstructive sleep apnea are:

- At close to **2.5 times** greater risk of developing breathing problems after surgery
- At more than **1.5 times** greater risk of developing heart problems after surgery

**Please indicate a response for the following questions:**

1. A sleep study is the best way to determine whether you have obstructive sleep apnea

YES  NO  Cannot Decide

1. People with obstructive sleep apnea may experience is excessive sleepiness/tiredness during the daytime.

YES  NO  Cannot Decide

1. Patients who undergo surgery with obstructive sleep apnea are at a higher risk of complications after surgery compared with patients who do not have obstructive sleep apnea.

YES  NO  Cannot Decide

**Consider the following Hypothetical Scenario and answer the questions below:**

You are to undergo planned surgery that is not urgent. This surgery will be done under anesthesia. *After performing an assessment, your doctor tells you that you are suspected of having obstructive sleep apnea.*

For patients undergoing surgery, having obstructive sleep apnea may increase the risk of complications after surgery. Measures available that may reduce these risks include a clear diagnosis of obstructive sleep apnea and proper management of the medical condition before surgery. Moreover, breathing will be monitored after surgery in case complications develop.

Your doctor gives you two options: 1) Delay surgery to undergo a sleep study and treatment to optimize your obstructive sleep apnea before surgery or 2) Proceed with surgery as planned. Receive monitoring of breathing after the surgery and be provided treatment if needed.

At the present, patients with obstructive sleep apnea develop more complications after surgery than patients without obstructive sleep apnea. As part of this research, it is important to know your perspectives on this issue.

1. **In light of this information, your physician would like to involve you in the decision making process and asks you which one of the following treatment options would YOU MOST prefer?** *(Select one)*

Method 1: *Proceed with the surgery* as planned. Receive monitoring of breathing after surgery and if needed be provided further treatment.

Method 2: *Delay surgery* to ensure my medical condition of obstructive sleep apnea is optimized before surgery. Receive monitoring for breathing after surgery and be provided further treatment if needed.

Method 3: I prefer to let my physician decide the course of action

******If you prefer “Method 1” or “Method 3”, proceed to question 3******

1. **If you prefer *“method 2”* (delay surgery), will you tolerate the following length of delay in order to undergo diagnosis and possible treatment to optimize your medical condition?**
2. 2 weeks delay:

YES  NO

1. 1 month delay:

YES  NO

1. 2 months delay:

YES  NO

1. **When making a decision as to which method of treatment you most prefer, how do you rate the importance of the following?**

*(Check off, with ‘1’ being Not Important and ‘5’ being Extremely Important)*

1. Shortest length of stay at the hospital:

1 2 3 4 5

*Not Important Extremely Important*

1. Lowest risk of complications during surgery:

1 2 3 4 5

*Not Important Extremely Important*

1. Lowest risk of complications after surgery:

1 2 3 4 5

*Not Important Extremely Important*

1. Lowest risk of being transferred to the Intensive Care Unit due to complications:

1 2 3 4 5

*Not Important Extremely Important*

1. Being involved in the decision-making process with my physician/physician asking me what I want:

1 2 3 4 5

*Not Important Extremely Important*

*(My Physician Decides) (Be completely involved/decide myself*
